# Transporting carriers for intracellular targeting delivery via non-endocytic uptake pathways

**DOI:** 10.1080/10717544.2017.1391889

**Published:** 2017-10-25

**Authors:** Zuhong Li, Yanhong Zhang, Danhua Zhu, Shuiqing Li, Xiaopeng Yu, Yalei Zhao, Xiaoxi Ouyang, Zhongyang Xie, Lanjuan Li

**Affiliations:** State Key Laboratory for Diagnosis and Treatment of Infectious Diseases, Collaborative Innovation Centre for Diagnosis and Treatment of Infectious Diseases, The First Affiliated Hospital, Zhejiang University School of Medicine, Hangzhou, PR China

**Keywords:** Transporting carriers, non-endocytic uptake, delivery systems, intracellular delivery, internalization mechanism

## Abstract

To develop novel therapies for clinical treatments, it increasingly depends on sophisticated delivery systems that facilitate the drugs entry into targeting cells. Profound understanding of cellular uptake routes for transporting carriers promotes the optimization of performance in drug delivery systems. Although endocytic pathway is the most important part of cellular uptake routes for many delivery systems, it suffers the trouble of enzymatic degradation of transporting carriers trapped in endosomes/lysosomes. Therefore, it is desirable to develop alternative transporting methods for delivery systems via non-endocytic pathways to achieve more effective intracellular delivery. In this review, we summarize the literature exploring transporting carriers that mediate intracellular delivery via non-endocytic pathways to present the current research status in this field. Cell-penetrating peptides, pH (low) insertion peptides, and nanoparticles are categorized to exhibit their ability to directly transport various cargos into cytoplasm via non-endocytic uptake in different cell lines. It is hoped that this review can spur the interesting on development of drug delivery systems via non-endocytic uptake pathway.

## Introduction

Rapid advance in biotechnology and nanotechnology have permitted the incorporation of therapeutic and imaging agents into a variety of delivery systems, offering new opportunities for treatment, detection, and prevention in clinical application (Jain & Stylianopoulos, 2010; Kratz & Warnecke, 2012; Gandhi et al., 2014). Many biologically active substance need to be delivered into intracellular compartments to exert their therapeutic effects in cytoplasm or other specific organelles, such as nucleus and mitochondria. However, the nature of biological membranes restricts the intracellular delivery of drug delivery systems. The cellular plasma membrane constitutes an effective barrier and regulates the transport of cargoes inside and outside the cell (Prestegard & Obrien, 1987; Jahn & Sudhof, 1999). Hence, the internalization pathway for entry into cells and the intracellular fate of transporting carriers are key issues for delivered cargoes to be efficient.

The internalization of drug delivery systems with their cargoes may utilize one or several physiological routes to enter into cells. In many cases, transporting carriers, such as nanoparticles (NPs), can be internalized via endocytosis after immobilization on the outer leaflet of plasma membrane. Endocytosis represents the natural route for macromolecules into cells (Mellman, 1996; D’Hondt et al., 2000; Doherty & McMahon, 2009). The plasma membrane locally invaginates or forms protrusions, surrounding, and enclosing the loaded substance. After membrane fission, the cargo molecules are located inside the lumen of the newly formed vesicles, mostly referred to as endosomes. Then, these vesicles are tethered to the next stop on their itinerary and fuse to their ultimate organelles, such as lysosomes. Although dozens of drug delivery systems can enter into cells via endocytosis, generally they are inevitably entrapped in endosomes and subsequent lysosomes, which are regarded as the overriding intracellular barriers for some payloads due to the harsh environment (Savic et al., 2006; Hillaireau & Couvreur, 2009). The lysosomes are the most acidic compartments in most cells, where low pH facilitates the activity of lysosomal hydrolases whose optimal pH ranging from 4.5 to 5.5, providing favorable conditions for enzymatic hydrolyses (Mellman et al., 1986). In this circumstance, many substrates become acid-denatured, and thus more susceptible to enzymatic degradation.

For some therapeutic agents, they have to be firstly delivered into the cytosol to become active drugs. Yet, the journey of transporting carriers entrapped inside endosomes and lysosomes results in degradation of the loaded therapeutic drugs. Accordingly, direct cytosolic delivery of cargo molecules via non-endocytic pathways is an optimal approach, and minimizes the degradation loss of transported agents. Therefore, it is desirable to develop new alternative delivery methods for enhanced non-endocytic uptake pathway to circumvent the entrapment in endosomes/lysosomes altogether. This review attempts to provide an overview of different transporting carriers currently known to be involved in non-endocytic uptake pathways for direct intracellular delivery. We hope that the review could be a valuable starting point for researchers who wish to investigate and develop delivery systems with the ability to transport cargo molecules via non-endocytic pathway.

## Uptake mechanism of transporting carriers

### Endocytic pathways

Endocytosis, the natural entry route for extracellular macromolecules into cell, encompasses a variety of mechanistically complex processes, which is still unveiling (Mellman, 1996). Eukaryotic cells display a number of distinct endocytic mechanisms. The processes of endocytosis play key roles in regulating mitosis, antigen presentation, and cell migration. They share some characteristics and effectors and may compensate for one another when one is abrogated. Several excellent reviews have presented an overview on this rapidly growing field (D’Hondt et al., 2000; Doherty & McMahon, 2009; Kumari et al., 2010; Iversen et al., 2011). Commonly the following main endocytic routes are distinguished: phagocytosis, clathrin-mediated endocytosis, caveolae-mediated endocytosis (CvME), and macropinocytosis.

#### Phagocytosis

Phagocytosis is a special type of endocytic pathway, which is defined as the engulfment of cells, bacteria, and large solid particles. It is typically restricted to specialized professional phagocytes such as macrophages, neutrophils, monocytes, and dendritic cells (Aderem & Underhill, 1999; Swanson, 2008). Phagocytosis is mediated by cup-like membrane extensions that are usually larger than 1 μm to internalize particles. It is noticed that the geometry, size, stiffness, and topography of the targeting particles all influence phagocytosis (Champion & Mitragotri, 2006; Underhill & Goodridge, 2012), which may give an impact on internalization of delivery systems by phagocytic cells. The phagosomes, containing opsonized complexes, will mature and fuse with lysosomes where the acidic environment and multiple enzymes promote degradation of internalized contents.

#### Clathrin-mediated endocytosis

Clathrin-mediated endocytosis is clathrin-mediated, receptor-dependent and GTPase dynamin-required endocytic pathway. In this pathway, a series of downstream events are activated after the recognition of ligands by receptors on the cell surface. The process comprises initiation of clathrin-coated pits, recruitment of cargo-specific proteins, coat assembly, dynamin-mediated scission, and uncoating by the ATPase. Then, the endocytotic vesicles are integrated into late endosomes and deliver their cargoes to lysosomes (Luzio et al., 2009).

#### Caveolae-mediated endocytosis

CvME is a type of cholesterol, dynamin-dependent, and receptor-mediated pathway (Nichols, 2003). CvME forms a special flask-shaped structure on the cell membrane called caveola, which is a plasma membrane domain enriched in cholesterol and sphingolipid (Parton & del Pozo, 2013). The fission of the caveolae from the cell membrane is mediated by the GTPase dynamin, and then generates the cytosolic caveolar vesicle (Hillaireau & Couvreur, 2009). The intracellular fate of caveolar vesicle remains under debate. It is worth noting that one research group draws the conclusion that the cavesome, a previously unrecognized membrane-bound organelle, itself is an artifact in cells over-expressing different constructs of caveolin-1 and that the term cavesome no longer should be used. This is especially important in light of CvME being regarded as a route away from lysosomal degradation. The caveolae that do pinch off are considered to fuse with normal acidified endosomes being able to transfer loaded cargos to lysosomes (Hayer et al., 2010; Parton & Howes, 2010; Iversen et al., 2011). Moreover, collected data from Kiss et al. support the discovery that sometimes the fusion of caveolar vesicle with lysosomes cannot be avoided (Kiss & Botos, 2009).

#### Macropinocytosis

Macropinocytosis, a type of distinct pathway, is typically characterized by the formation of membrane ruffles and the engulfment of large volumes of fluid into large uncoated vacuoles, known as macropinosomes (Sarkar et al., 2005; Kerr & Teasdale, 2009). Macropinosomes form spontaneously or in response to growth factors or other signals (Kaplan et al., 2005). Their formation can be also induced by bacteria, apoptotic bodies, and viruses (Mercer & Helenius, 2009). Distinct from clathrin-coated vesicles and caveolae, the macropinosomes have no apparent coat structures and are heterogenous in vesicular size distribution (Hewlett et al., 1994). Macropinosomes mature by modification of their lipid and regulatory membrane protein composition, before fusion with the degradative compartments of the cells.

### Non-endocytic pathways

As the most important part of cellular uptake routes, the process of endocytic pathway has received a great deal of attention in the last couple of years, especially for clathrin-mediated endocytosis. The mechanisms that are involved in the process of recruiting cargo into developing pits and subsequently forming vesicles are becoming increasingly understood. In contrast, there have been, so far, a few of studies concerning on non-endocytic uptake pathway, because this pathway is usually poorly considered as it challenges the idea of non-permeability of membranes to large hydrophilic molecules (Jahn & Sudhof, 1999). However, the discovery of cell-penetrating peptides (CPPs) and some NPs capable of direct cytosolic delivery reminds that there are naturally existing non-endocytic pathways, although a tremendous amount of research work is needed to reveal the specific details behind this mechanism. In the following contents, available articles covering the transporting carriers for non-endocytic pathway are summarized and classified into two categories to discuss.

## CPPs and pHLIPs-mediated non-endocytic uptake

### Cell-penetrating peptides

CPPs, also referred as protein transduction domains, are a class of diverse peptides typically with 5–30 amino acids, which derived from natural proteins and synthetic peptides (Bechara & Sagan, 2013; Shi et al., 2014). Commonly, based on their physical-chemical properties, CPPs can be classified into three subgroups (Milletti, 2012): cationic CPPs, amphipathic CPPs, and hydrophobic CPPs. Since the two first CPPs, Tat (Green et al., 1989; Vives et al., 1997) and Penetratin (Derossi et al., 1994) originated from transactivating protein of HIV-1 and Drosophila homeoprotein, respectively, were discovered more than 20 years ago, CPPs have been reported in a number of applications (Gupta et al., 2005; Bolhassani, 2011). CPPs serving as transporting carriers have been employed to deliver various cargoes including proteins, peptides, nucleic acids, and contrast agents into cells *in vitro* and *in vivo*.

Quantitation of the membrane translocation efficiency of cationic oligopeptides was determined, allowing for selective measurement of direct translocation (Zaro & Shen, 2003). It was found that Tat-(YGRKKRRQRRR), YG(R)_9_ and guanidinated-YG(K)_9_ were preferentially translocate into the cytosolic compartment, while YG(K)_9_ was primarily endocytosed in Chinese hamster ovary cells. Studies of various oligoarginine peptides (4–15 residues) demonstrated that internalization through direct translocation remained constant, while the amount internalized via endocytosis increased with arginine length, indicating that oligopeptide translocation requires the guanidine structure of arginine, while endocytosis depends only on the number of positive charges. Moreover, direct translocation of YG(R)_9_ was not affected by incubation at 16 °C as compared to that at 37 °C, and only partially inhibited at 4 °C in Chinese hamster ovary cells (Zaro & Shen, 2005). Membrane translocation was not inhibited to the same extend as endocytosis following treatment with ammonium chloride, hypertonic medium, amiloride, or filipin. Intracellular trafficking of cargos attached nona-arginine (GLPK(FITC)RRRRRRRRR) were measured in four cancer cell lines with a series of microenvironments altered by the presence of endocytic inhibitors and different temperature (Ma et al., 2011). The results revealed that FITC-CPPs may enter cells rapidly via direct translocation in addition to the endocytic route, while Avidin-CPPs tend to be internalized by macropinocytosis in an energy-dependent manner with slower rates. Kinetics of translocation into cells with NrTPs (YKQCHKKGGKKGSG) in lymphocyte and monocyte cell was addressed (Rodrigues et al., 2013). Uptake results obtained at 4 °C or using chemical endocytosis inhibitors support the importance of non-endocytic mechanisms in the cellular internalization of NrTPs. Fretz et al. analyzed the effects of temperature, concentration, and plasma membrane cholesterol levels on the uptake of Alexa Fluor 488 attached CPPs (R8) in KG1a cells (Fretz et al., 2007). The results found that Alexa Fluor 488-R8 uniformly labels the cytoplasm and nucleus at 4–12 °C, suggesting a pathway of direct translocation across the plasma membrane. Relatively small increases in peptide concentration or sequestering plasma membrane cholesterol can enhance the fraction of the peptide that localizes to the cytosol. These processes seemed to be specific for the peptide, as they did not observe any parallel increases in membrane permeability or toxicity.

Mass spectrometry-based method was utilized to quantify the internalization of biotin-conjugated Tat peptide (Biot-G_4_-RKKRRQRRRPPQ) and R_9_ (Biot-G_4_-R_9_) at 37 and 4 °C in both wild type and proteoglycan-deficient Chinese hamster ovary cells (Jiao et al., 2009). Both direct translocation and endocytosis were internalization pathways for Biot-CPPs. Direct translocation occurred at low extracellular peptide concentration, whereas endocytosis was activated at higher concentrations. Direct translocation operates in a narrow time window, which implied a specific lipid/peptide co-import in cells. Zhang et al. developed stearic acid modified arginine octamer derivatives (SAR_6_EW) for the enhanced transport of insulin (Zhang et al., 2015). A significant decrease in the fluorescent intensity was observed when the cells were treated with SAR_6_EW-insulin at 4 °C. However, there were still fluorescent signals achieved in treated cells, suggesting that both energy dependent pathway and energy-independent direct translocation were involved, which were consistent with the results of inhibitor studies. The protein uptake and translocation efficiency of four avidin linked CPPs (Penetratin, Tat peptide, Transportan, and pVEC) were studied in HeLa cells (Saalik et al., 2004). The results demonstrated that even under conditions of cellular energy depletion, ceasing of cellular traffic, and partial depolarization of plasma membrane, peptide-protein complexes still entered into cells, suggesting an energy, and endocytosis independent internalization pathway. Pan et al. evaluated the internalization process of designed CPPs, STR-KV (stearylation-HHHKKKVVVVVV), complexed with siRNA targeting at the *glyceraldehyde-3-phosphate dehydrogenase* (GAPDH) gene in A549 and CHO-K1 cells (Pan et al., 2016). It was elucidated that the electrostatic interaction of STR-KV/siRNA complex with heparin sulfate proteoglycans at the cell membrane surface triggered the energy-independent uptake of the majority of the complexes, using heparin treatment, and chemical endocytic inhibitors. Intracellular trafficking and internalization kinetics observed by confocal microscopy confirmed that the complex was uptaken through a non-endocytic pathway.

**Figure 1.**
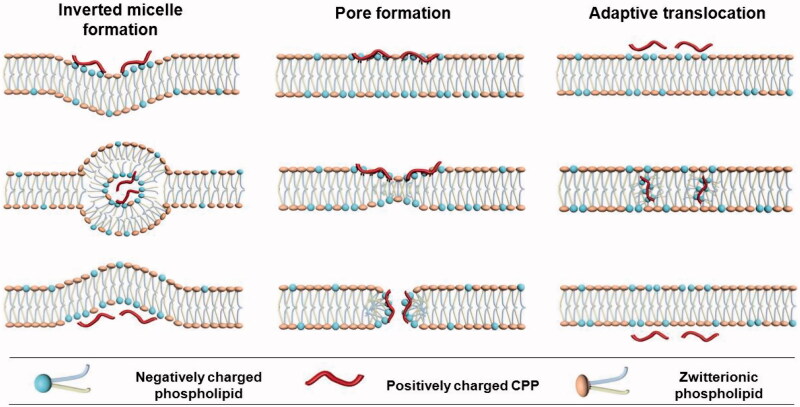
The proposed internalization mechanisms of direct translocation for positively charged CPPs.

Direct translocation involves destabilization of the plasma membrane in an energy- and temperature-independent manner. Some hypotheses, including inverted micelle formation (Derossi et al., 1996), pore formation (Matsuzaki et al., 1996), adaptive translocation (Rothbard et al., 2004), the carpet-like model (Pouny et al., 1992), and the membrane thinning model (Lee et al., 2005), were proposed to explain direct translocation of CPPs across the lipid bilayer (Figure 1). The first step in all these mechanisms comprises interaction of the positively charged CPP with negatively charged components of membrane, such as phospholipid bilayer and heparin. Peptide folding and transient destabilization of lipid membrane are involved in this process. The subsequent steps of internalization depend highly on the peptide concentration, peptide sequence and lipid composition in each model.

The inverted micelle model was proposed for the direct translocation of penetratin (Derossi et al., 1998; Alves et al., 2008; Joanne et al., 2009; Kawamoto et al., 2011). In addition to the interaction between the positively charged CPP and negatively charged lipid components, interaction between hydrophobic residues, such as tryptophan and the hydrophobic part of the membrane is also shown to be involved in this mechanism. Pore formation model consists of barrel stave model and toroidal model (Deshayes et al., 2006; Herce & Garcia, 2007; Herce et al., 2009). The barrel stave model shows that helical CPPs form a barrel by which hydrophobic residues are close to the lipid chains, and hydrophilic residues construct the central pore. In the toroidal model, lipids bend in a way that the CPP is always close to the headgroup, both CPP and lipids forming a pore. In both models, pores appear when the peptide concentration is beyond a certain concentration threshold, which is varied for different CPPs. The adaptive translocation model proposes that oligoarginines can possess either a hydrophilic or hydrophobic character depending on the associated counteranion due to the capacity of guanidinium headgroups to form bidentate hydrogen bonds (Wender et al., 2008). Then, the interaction between guanidinium-rich peptides and the phosphate lipid headgroups will mask the peptide charge, attenuating its polarity and enabling its diffusion into and across the plasma membrane.

### pH (Low) insertion peptides

**Figure 2.**
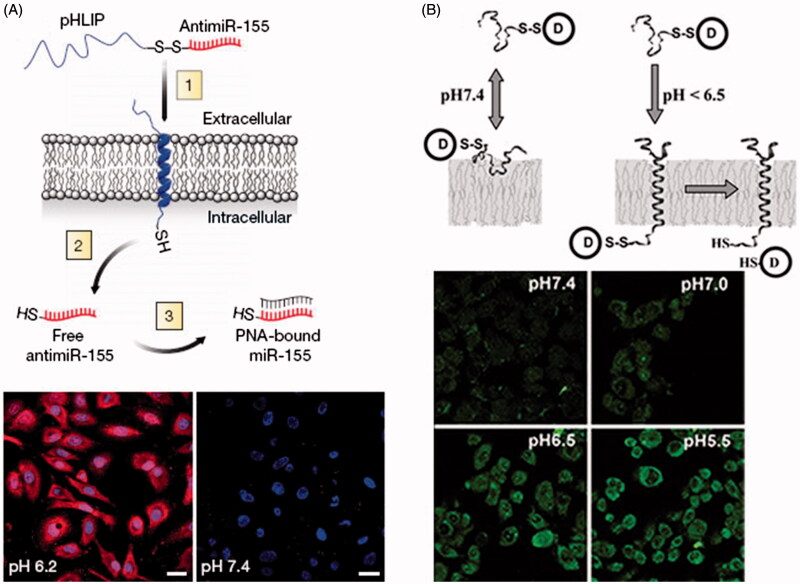
Direct cytosolic cargos delivery mediated by pHLIP. (A) Schematic of pHLIP-mediated PNA antimiR delivery and confocal projection of A549 cells incubated with labeled pHLIP-antimiR at different pH (Cheng et al., 2015). Reproduced with permission. Copyright 2015, Nature Publishing Group. (B) Schematic diagram of pHLIP for cargo delivery into cell and fluorescence images of Hela cells incubated with cleavable pHLIP-S-S-dansyl construct at different pH value (Reshetnyak et al., 2006). Reproduced with permission. Copyright 2006, National Academy of Sciences.

pH (Low) insertion peptides (pHLIPs) are water soluble and moderately hydrophobic polypeptides originally derived from the bacteriorhodopsin C helix. At neutral pH, pHLIP is in equilibrium between soluble and membrane-bound unstructured forms, whereas in a low pH environment, a pHLIP is triggered by acidity to fold and insert across a membrane to form a stable trans-membrane α-helix (Hunt et al., 1997; Andreev et al., 2009; Musial-Siwek et al., 2010). It is shown that the N terminus of pHLIP stays outside of the bilayer, while the C terminus inserts across the lipid bilayer (Reshetnyak et al., 2007). The insertion of pHLIP into lipid bilayer is associated with an energy release which can be used to move cargo molecules across a membrane, raising the possibility of transporting drug into cell cytoplasm (Karabadzhak et al., 2012). It is noting that pHLIP-mediated translocation of cargo molecules into cells is not mediated by endocytosis or interactions with cell surface receptors (Figure 2).

Peptide nucleic acid (PNA) can inhibit both transcription and translation of gene to which it has been targeted, which holds promise for its use for antigene and antisense therapy (Nielsen et al., 1991; Ray & Norden, 2000; Kaihatsu et al., 2004). However, the major obstacle is the delivery of PNA across the membrane into cytoplasm. Reshetnyak et al. examined the ability of pHLIP to translocate a fluorescent-labeled 12-base PNA (TAMRA-CATAGTATAAGT-Cys) into HeLa cells (Reshetnyak et al., 2006). After incubation of pHLIP-S-S-PNA-TAMRA with HeLa cells, no retention of fluorescence was seen in the cells at normal pH, but significant cytoplasm fluorescent staining of cells was observed at low pH value. By contrast, the treatment of cells with PNA-TAMRA did not show fluorescent staining of cells at pH 6.5 or 7.4. Moreover, they prepared pHLIP conjugated to dansyl dye Ph–TRITC via a disulfide bond, and tested the construct with live HeLa cells at different pH values. The results showed that the uptake of dansyl dye was significantly higher at low pH. The relative uptake at pH 6.5 was about 4.3-fold higher than that at pH 7.4. pHLIP was also employed to translocate phalloidin, a cell impermeable polar toxin, to inhibit the proliferation of Hela, JC, and M4A4 cancer cells in a pH-dependent fashion (An et al., 2010; Wijesinghe et al., 2011). The polypeptide complex carried phalloidin attaching to the C terminus via a disulfide bond that was cleaved inside cell cytoplasm to release the toxin. Thevenin et al. (2009) studied the properties of cell impermeable cargo molecules that can be delivered into cell cytoplasm by pHLIP in cancer cells. Four cyclic hexapeptides were selected as model cargo molecules, which contain four X positions that were varied as Ser, Asp, Asn, or Arg residues (NC(X)_4_), allowing adjustments of cargo polarity. The results indicated that NC(Ser)_4_ and NC(Asp)_4_ cargos were translocated and released into cell cytoplasm only when the cells were incubated at pH 6.2. These findings in the cancer cells correlated very well with that obtained with the lipid vesicles, which supported that pHLIP peptides traversed cell membrane via direct insertion into the bilayer, reinforcing the view of pHLIP-assisted translocation independent of membrane protein or endocytosis. Then, they developed a pHLIP-based delivery platform that targeted the acidic tumor microenvironment to evade systemic clearance by the liver and facilitated cell entry via a non-endocytic pathway (Cheng et al., 2015). The attachment of antimiRNAs to pHLIP produced a novel construct that could transport antimiRNAs across plasma membranes under acidic conditions in solid tumors (pH approximately 6), and effectively inhibit the miR-155 oncomiR in a mouse model of lymphoma.

In contrast to CPPs, pHLIPs still stay in the cellular membrane after insertion, translocating one end into cytoplasm and leaving the other end in the extracellular environment. Thus, pHLIP possesses dual delivery capabilities, which can tether cargo molecules to cellular surfaces and/or can translocate and release cell impermeable molecules into cytoplasm. Usually, polar cargo molecule attaching to the C-terminus of pHLIP utilizes a chemical bond that is stable outside the cellular environment, but cleaved inside the cytoplasm. The chemical conjugation of polar molecules to pHLIPs is convenient, since Lys and Cys residues can be easily introduced in the synthesis of polypeptides.

## Nanoparticle mediated non-endocytic uptake

**Figure 3.**
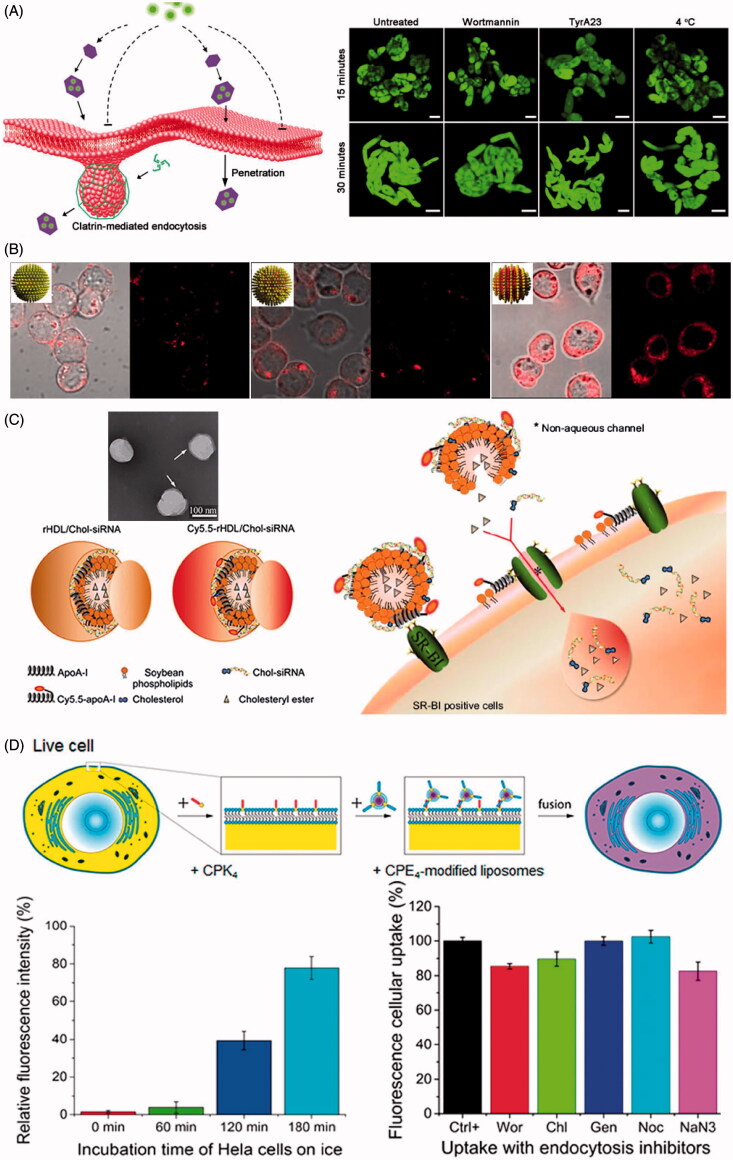
Nanoparticle mediated intracellular delivery via non-endocytic uptake. (A) Schematic diagram of LDH-Lactate-NS internalization via free penetration and fluorescence images of BY-2 cells incubated with LDH-lactate-NS-TRITC under different conditions (Bao et al., 2016). Reproduced with permission. Copyright 2016, Nature Publishing Group. (B) Fluorescence images of dendritic cells incubated with surface functional gold nanoparticles at 4 °C (Verma et al., 2008). Reproduced with permission. Copyright 2008, Nature Publishing Group. (C) Proposed mechanism for SR-BI mediated cytosolic delivery of FAM-Chol-siRNA in Cy5.5-rHDL/FAM-Chol-siRNA complexes (Ding et al., 2014). Reproduced with permission. Copyright 2014, Elsevier. (D) Scheme of fusion between cell and liposomes as well as the effect of ice incubation and endocytic inhibitors on delivery of fluorescent dyes by liposomes to Hela cells (Yang et al., 2016). Reproduced with permission. Copyright 2016, American Chemical Society.

NPs offer new opportunity to improve the delivery of various drugs into cells (Giljohann & Mirkin, 2009; Riehemann et al., 2009; Jain & Stylianopoulos, 2010; Dreaden et al., 2012). Previous studies have demonstrated that different endocytic mechanisms involved in the cellular internalization of NPs, which dramatically influenced by physicochemical properties of NPs, such as size, surface charge and composition (Gratton et al., 2008; Harush-Frenkel et al., 2008; Mailander & Landfester, 2009; Ernsting et al., 2013). Recent updates of knowledge about uptake pathways imply that NPs can pass through cell membrane and entry into cytoplasm via non-endocytic pathway (Figure 3).

Bao et al. reported that the positively charged delaminated layered double hydroxide lactate nanosheets (LDH-lactate-NS) with 0.5–2 nm thickness and 30–60 nm diameter could enter the cytoplasma in terms of free penetration of plasma membrane in BY-2 cell line (Bao et al., 2016). The LDH-lactate-NS electrostatically binding negative charged fluorescent dyes or ssDNA to form neutral nano-aggregates were internalized into cytosol via non-endocytic pathway without uptake inhibition of low temperature and inhibitor treatment. Bossi et al. found that cobalt oxide NPs, but neither cobalt nor cobalt oxide NPs surrounded by protein corona, can enter inside the cells by penetrating plasma membranes (Bossi et al., 2016). Mature Xenopus oocytes were filled with Calcein, whose fluorescence was strongly quenched by divalent metal ions, and exposed to different NPs to quantify quenching, indicating the increase of Co^2+^ concentration released from cobalt NPs located in the cytosol. The results suggested that Co_3_O_4_ NPs succeed in penetrating the plasma membrane and caused the observed quenching activity in cytoplasm. Quenching experiments under the condition of Dynasore pre-incubation showed that the internalization of Co_3_O_4_ NPs was dynamin-independent process. Fully grown oocytes exposed to Lucifer yellow and Co_3_O_4_ NPs demonstrated that there was no formation of endocytotic vesicles after Co_3_O_4_ NPs exposure. One interesting study stated that 6 nm gold NPs coated with subnanometer striations of alternating anionic and hydrophobic groups can directly penetrate the plasma membrane into cytoplasm via energy-independent pathway without bilayer lipid disruption in dendritic cells (Verma et al., 2008). Those NPs were modified with a shell of hydrophobic and anionic ligands regularly arranged in ribbon-like domains of alternating composition. Under the temperature of 4 °C, the ‘striped’ NPs still entered cells at substantial levels, exhibiting a diffuse fluorescence pattern consistent with cytosolic location. Further test in the presence of pharmacological inhibitors of sodium azide and 2-deoxyglucose showed that ‘striped’ NPs readily entered the cytoplasm under the condition of inhibited active uptake processes.

A research group reported that amino functionalized cross-linked polystyrene microspheres with diameters of 0.2, 0.5, and 2 μm were internalized via an energy-independent non-endocytic pathway in mouse melanoma cells (Alexander et al., 2010). The uptake of microsphere was independent of both ATP and cholesterol depletion, which were both required for endocytosis. Utilization of a number of inhibitors to examine endocytosis had no impact on microsphere uptake, meanwhile lysosomal, and endosomal tracking agents did not show co-localization of microsphere with lysosomes/endosomes. In another study, arginine analogs conjugated mesoporous silica nanoparticles (MSN) (300 nm) enhanced the uptake through non-endocytotic pathway by introducing Arg analogs with varying side chain lengths in HeLa cells (Wu et al., 2013). After cells were treated with sodium azide and 2-deoxy-D-glucose, 20% of the cells still showed uptake of MSN upon inhibition of endocytosis, suggesting portion of the MSN crossed the cell membrane through energy-independent non-endocytotic pathways. Mu et al. found that amorphous silica NPs with 14 nm diameters can directly enter into A549 cells in the absence of serum proteins (Mu et al., 2012). Transmission electron microscopy and energy-dispersive X-ray spectroscopy demonstrated that silica NPs entered into cytoplasm but not within membrane bound vesicles or in the nucleus.

A novel nanoparticle was described for direct drug delivery into the cytosol of live cells *in vitro* and *in vivo* utilizing membrane fusion between liposomes and cells (Yang et al., 2016). A pair of complementary coiled-coil lipopeptides (CPK_4_ and CPE_4_) was integrated in the cellular membranes and lipid bilayer of liposomes respectively, which resulted in targeted membrane fusion with concomitant release of encapsulated cargoes including fluorescent dyes and doxorubicin entrapped in liposomes. Utilization of a wide variety of endosome trackers and endocytosis inhibitors demonstrated that the dominant pathway for intracellular delivery is membrane fusion between liposomes and plasma membrane of live cells. Torchilin et al. reported that TAT peptides attached liposomes of 200 nm diameters can be directly translocated into cell cytoplasm via energy-independent process in H9C2 cells and BT20 cells (Torchilin et al., 2001). The internalization of TAT-liposomes continued effectively under 4 °C treatment, suggesting the translocation process seemed to be energy-independent. Moreover, the internalized process was not inhibited by sodium azide or IAA, which indicated that there was no involvement of oxidative respiration or cytoskeleton. Moreover, one study employed reconstituted high density lipoprotein to load cholesterol-siRNA (rHDL/Chol-siRNA complex), providing an effective approach to directly transfer Chol-siRNA into the cytoplasm via scavenger receptor BІ-mediated non-endocytotic mechanism (Ding et al., 2014). The cellular uptake of Chol-siRNA in SR-BІ high (MCF-7) and low-expressing (HT1080) cell line suggested that the delivery of Chol-siRNA in rHDL was mediated by scavenger receptor BІ. Co-localization of stained lysosomes and FAM-Chol-siRNA showed that FAM-Chol-siRNA appeared as a direct cytosolic delivery with endocytosis-independent process. Meanwhile, the observation of Cy5.5-rHDL/FAM-Chol-siRNA internalization indicated that the uptake process did not only arise from bringing the SR-BІ-docked rHDL/Chol-siRNA complex close to the plasma membrane, but it would involve permitting the removal of Chol-siRNA from the complex and selective absorption into cytosol.

As far as is known, many factors are involved in the selection of uptake pathway in endocytosis, such as particle size and shape, charge density, cell types, and even culture condition (Alexis et al., 2008; Jiang et al., 2008; Xiang et al., 2012). Also, these factors influence the uptake pathways for nanoparticle-mediated non-endocytosis. However, in some cases, NPs with different size distribution, surface charge, and composition seem to be all capable of directly penetrating cells following the same mode. For instance, the investigation of polystyrene microspheres with differing diameters of 200 nm, 500 nm, and 2 μm indicated that the direct penetration of three NPs was energy-independent process via a rapid non-endocytic pathway. While the volume of formed endocytic vesicle after engulfment in endocytosis usually limits the size distribution of transporting carriers in specific endocytic pathway, typically a diameter of 120 nm for clathrin pit and a diameter of 60–80 nm for caveolae. Further, neutral LDH-nanosheet conjugates, consisting of negatively charged biomolecules and positively charged layered double hydroxide lactate nanosheets with a 0.5–2 nm thickness and a 30–60 nm diameter, also exhibited direct penetration following an energy-independent non-endocytic pathway. Nevertheless, to fully explore the nanoparticle-mediated non-endocytosis, there is actually a lack of comprehensive study to characterize the uptake pathways in some studies which roughly confirm the appearance of non-endocytic uptake to exclude the endocytosis. Thus, it is strongly recommended that more quantitative analysis should be employed to carefully examine and validate the process of non-endocytic pathway, avoiding the disturbance of artifacts arising from endocytosis (Skotland et al., 2015).

## Methods used to study non-endocytic uptake

Currently there is no well-established general methodology on how non-endocytic uptake of transporting carriers should be studied. The adoption of different methods to exclude presently known endocytic pathways serves as primary way to characterize non-endocytic uptake pathways. Exclusion studies can be performed using pharmacological inhibitors, molecular probes, and organelle specific dyes.

Pharmacological inhibitors are the effective tools to block specific endocytic pathway in order to determine whether it plays an important role in the uptake of transporting carriers. However, none of the commonly used inhibitors of endocytosis is absolutely specific. Besides their cell type-dependent manner, they can either affect the actin cytoskeleton with side effects or interfere with alternative endocytic pathways simultaneously.

To distinguish phagocytic and macropinocytic pathways with non-endocytic uptake pathways, some pharmacological inhibitors are utilized in mechanism studies, including inhibitor of sodium-proton exchange ‘amiloride’, F-actin depolymerizing drug ‘cytochalasin D’, and inhibitor of phosphoinositide metabolism ‘wortmannin’. The specificity of all these inhibitors is still in doubt as depolymerizing F-actin and inhibition of phosphoinositide metabolism may also disrupt other endocytic pathways. For example, cytochalasin D is also applied as inhibitor in the study of CvME (Parton et al., 1994). Methods such as incubation of cells with hypertonic sucrose and inhibitor ‘chlorpromazine’ were used to block clathrin-dependent endocytosis and to demonstrate the existence of different uptake mechanisms. However, the co-incubation with hypertonic sucrose is unspecific method with side-effects on cellular physiology. For instance, high sucrose can also affect other types of endocytosis than clathrin-mediated, since the fluid phase uptake can be completely blocked in fibroblasts (Heuser & Anderson, 1989). As to caveolae-mediated endocytic pathway, the commonly used inhibitors are methyl-β-cyclodextrin (MβCD), filipin, nystatin, and genestein. MβCD causes acute cholesterol depletion to block caveolae-dependent endocytosis. However, cholesterol is not only important for the caveolae-mediated uptake, but also for other mechanism such as macropinocytosis and clathrin-dependent uptake (Van Kerkhof et al., 2001; Grimmer et al., 2002). Furthermore, the most direct way to distinguish endocytic pathways and non-endocytic pathways is the utilization of inhibitor or method for energy depletion, because most endocytic uptake is energy-dependent active transport process. Sodium azide (an ATPase inhibitor) and low temperature (4 °C) are commonly used inhibitor and method. In some conditions, low temperature and ATPase inhibitor should be used together because some of the non-endocytic pathways are also sensitive to low temperature (Pan et al., 2016).

Except for pharmacological inhibitors, molecular probes are also important tools for the studies in non-endocytic uptake pathways. Transferrin is often used as a probe of clathrin-mediated endocytosis in many studies (Alexander et al., 2010; Pan et al., 2016). Transferrin receptor mediates transferrin uptake via clathrin-mediated endocytosis, so that it can be used as specific marker and detected by anti-transferrin receptor. The organelle specific dyes are other ideal tools for the detection of co-localization. LysoTracker is the widely used dye for lysosomes. Combined with confocal imaging technology, the co-localization of labeled transporting carriers, and intracellular compartments can be observed visually. Measuring the co-localization with specific organelle dyes can be very helpful, but it should be kept in mind that apparent co-localization may be obtained from structures in close proximity without real co-localization in the same organelle. Moreover, false co-localization can easily be obtained if one fluorescent marker displays large patches or continuous areas of fluorescence which inevitably will overlap with fluorescent spots of other markers (Iversen et al., 2011).

## Conclusions and perspectives

In recent years, the field of endocytosis has undergone enormous growth with the realization that these internalization pathways play important roles in the regulation of extracellular nutrients uptake, membrane dynamics, and recycling cellular components. Meanwhile, a number of drug delivery systems have been proved to enter cells via endocytosis, albeit they typically suffer the harsh environment of endosomes and lysosomes during intracellular trafficking. In some cases, the delivered therapeutic agents can keep their pharmacological activities after endocytosis processes, however, many macromolecular drugs such as polypeptides, proteins, and nucleic acids are susceptible to enzymatic degradation and lose their activities after entrapment in endosomes and lysosomes. Thus, it is highly desirable to develop alternative delivery methods that bypass the endocytic pathways. Here, CPPs, pHLIPs, and some NPs are reviewed to exhibit their abilities to directly transport the cargoes into cytoplasm via non-endocytic pathways (Table 1). Based on pH-dependent membrane-associated folding, pHLIPs can fold and insert across cell membranes to deliver C-terminus linked cargo molecules into cytosol at low pH value. Also, different length and sequence of CPPs conjugated with small molecules, protein, and siRNA can enter into cytoplasm in the manner of direct translocation in different cell lines. In addition, some NPs with distinct diameter, composition, and surface properties could be internalized into cell via energy-independent non-endocytic pathways rather than endocytosis.

**Table 1.** Transporting carriers mediated non-endocytic uptake.

Transporting carrier	Cargo	Uptake mechanism	Cell line	Ref.
GLPKRRRRRRRRR	FITC	Direct translocation	MCF-7, MDA-MB-231	Ma et al. (2011)
R8: RRRRRRRR	Alexa Fluor 488	Direct translocation	KG1a	Fretz et al. (2007)
NrTP: YKQCHKKGGKKG -SG	Rhodamine B	Direct translocation	Lymphocyte, monocyte	Rodrigues et al. (2013)
Tat: G4-RKKRRQRRRPPQ, R9:G4-RRRRRRRRR	Biotin	Direct translocation	CHO-K1, CHO-pgsA-745	Jiao et al. (2009)
Stearylation-HHHKKKVVVV-VV	siRNA	Direct translocation	A549, CHO-K1	Pan et al. (2016)
pHLIPs: AAEQNPIYWARYA- DWLFTTPLLLLDLALLVDA-DEGTCG	Peptide nucleic acid (PNA),Ph-TRITC	Trans-membrane insertion	HeLa, CRL-2730, CRL-2116	Reshetnyak et al. (2006)
	Phalloidin	Trans-membrane insertion	Hela, JC, M4A4	An et al. (2010)
	NC(Ser)_4_, NC(Asp)_4_	Trans-membrane insertion	HeLa	Thevenin et al. (2009)
	PNA antimiRNAs	Trans-membrane insertion	A549	Cheng et al. (2015)
Gold nanoparticles	BODIPY dye	Unclear	DC2.4	Verma et al. (2008)
LDH-lactate nanosheets	ssDNA	Unclear	BY-2	Bao et al. (2016)
Polystyrene microspheres	Cy5	Unclear	HeLa, K625	Alexander et al. (2010)
Liposomes	PI, DOX	Membrane fusion	HeLa, CHO, 3T3	Yang et al. (2016)
High density lipoprotein nanoparticles	Chol-siRNA	SR-BІ mediated non-endocytosis	MCF-7	Ding et al. (2014)

Although recent studies bring strong data for the existence of direct translocation for CPPs, the mechanisms underlying the non-endocytic uptake of CPPs remain far from being fully understood. To date, several hypotheses have been proposed to explain direct translocation of CPPs across the lipid bilayer, including inverted micelle formation, pore formation, adaptive translocation, the carpet-like model, and the membrane thinning model. The model of inverted micelle formation was first proposed to explain the direct translocation of penetratin. Molecular dynamic simulation and^31^ P-NMR experiments emphasize the importance of tryptophan residues and hydrogen bonding between the guanidinium headgroups and phosphate groups. Pore formation model was evoked to illustrate the passive diffusion of Tat and arginine-rich peptides across the plasma membrane with molecular dynamic simulations and electrophysiology experiments. Studies with other CPPs also suggested the formation of transient pores as a mechanism of direct translocation. While these models provide reasonable explanation of molecular mechanism for direct translocation of CPPs, the exact mechanisms of non-endocytic uptake is still indecisive. In addition, due to their cationic nature, the potential toxicity of CPPs to cells or tissues needs to be comprehensively investigated *in vitro* and *in vivo* if they are to be utilized as drug delivery systems. The *in vitro* toxicity of CPPs has been detected frequently, but *in vivo* evaluations are limited currently. Generally, toxicity of CPPs mainly bases on two patterns: toxic effect on lipid membrane of cells and organelles, and specific interaction with cell ingredients (Ziegler, 2008). Exposure time, length, and concentration of CPPs, attached cargoes and cell types can influence their toxicity (El-Andaloussi et al., 2007).

Inspired by the SNARE protein complex, an artificial membrane fusion system composed of a complementary pair of lipidated coiled-coil peptides was developed, enabling targeted liposome-cell membrane fusion. The endocytic pathways are almost completely circumvented, and the major site of cargo release is at the plasma membrane. Additionally, the rHDL/Chol-siRNA NPs were prepared to provide a highly effective approach to directly transport Chol-siRNA into cytoplasm via the SR-BI mediated non-endocytic mechanism. The recognition of apoA-I by SR-BI would result in formation of a non-aqueous ‘channel’ and subsequent cross-membrane transfer of Chol-siRNA cargo from rHDL to cytosolic compartments without internalization of the intact rHDL/Chol-siRNA complexes. Yet, the mechanism for direct penetration of NPs is still unclear. There have no models or hypotheses were proposed to explain this energy-independent non-endocytic pathway.

Molecular and functional dissection of non-endocytic mechanisms is vital for rational design of highly potential drug delivery systems to directly transport cargos into cytoplasm. The investigations are now underway regarding what are the structural bases of transporting carriers for non-endocytic uptake, how do they interact with the cellular components to drive their way across the membrane, and whether certain non-endocytic pathway is found only in specialized cell type. Furthermore, another big question in this field is how the non-endocytic pathways are regulated with other internalization processes such as endocytosis. Endocytosis is highly active process existing in all cell types. Several endocytic pathways are rather unspecific, therefore, it is highly unlikely that any transporting carrier would enter cell exclusively via non-endocytic pathways. What is the balance between endocytic/non-endocytic pathways? Which factors would affect this balance? Addressing these important issues is the major challenge for future studies.

## References

[CIT0001] Aderem A, Underhill DM. (1999). Mechanisms of phagocytosis in macrophages. Annu Rev Immunol 17:593–623. 10358769 10.1146/annurev.immunol.17.1.593

[CIT0002] Alexander LM, Pernagallo S, Livigni A, et al. (2010). Investigation of microsphere-mediated cellular delivery by chemical, microscopic and gene expression analysis. Mol Biosyst 6:399–409. 20094660 10.1039/b914428e

[CIT0003] Alexis F, Pridgen E, Molnar LK, Farokhzad OC. (2008). Factors affecting the clearance and biodistribution of polymeric nanoparticles. Mol Pharm 5:505–15. 18672949 10.1021/mp800051m PMC2663893

[CIT0004] Alves ID, Goasdoue N, Correia I, et al. (2008). Membrane interaction and perturbation mechanisms induced by two cationic cell penetrating peptides with distinct charge distribution. Biochim Biophys Acta. 1780:948–59. 18498774 10.1016/j.bbagen.2008.04.004

[CIT0005] An M, Wijesinghe D, Andreev OA, et al. (2010). pH-(low)-insertion-peptide (pHLIP) translocation of membrane impermeable phalloidin toxin inhibits cancer cell proliferation. Proc Natl Acad Sci USA. 107:20246–50. 21048084 10.1073/pnas.1014403107 PMC2996653

[CIT0006] Andreev OA, Engelman DM, Reshetnyak YK. (2009). Targeting acidic diseased tissue: new technology based on use of the pH (Low) insertion peptide (pHLIP). Chim Oggi 27:34–7. 20037661 PMC2796806

[CIT0007] Bao WL, Wang JY, Wang Q, et al. (2016). Layered double hydroxide nanotransporter for molecule delivery to intact plant cells. Sci Rep 6:26738. 27221055 10.1038/srep26738 PMC4879670

[CIT0008] Bechara C, Sagan S. (2013). Cell-penetrating peptides: 20 years later, where do we stand? FEBS Lett 587:1693–702. 23669356 10.1016/j.febslet.2013.04.031

[CIT0009] Bolhassani A. (2011). Potential efficacy of cell-penetrating peptides for nucleic acid and drug delivery in cancer. Biochimi Biophys Acta 1816:232–46. 10.1016/j.bbcan.2011.07.006 21840374

[CIT0010] Bossi E, Zanella D, Gornati R, Bernardini G. (2016). Cobalt oxide nanoparticles can enter inside the cells by crossing plasma membranes. Sci Rep 6:22254. 26924527 10.1038/srep22254 PMC4770291

[CIT0011] Champion JA, Mitragotri S. (2006). Role of target geometry in phagocytosis. Proc Natl Acad Sci USA 103:4930–4. 16549762 10.1073/pnas.0600997103 PMC1458772

[CIT0012] Cheng CJ, Bahal R, Babar IA, et al. (2015). MicroRNA silencing for cancer therapy targeted to the tumour microenvironment. Nature 518:107–10. 25409146 10.1038/nature13905 PMC4367962

[CIT0013] D'hondt K, Heese-Peck A, Riezman H. (2000). Protein and lipid requirements for endocytosis. Annu Rev Genet 34:255–95. 11092829 10.1146/annurev.genet.34.1.255

[CIT0014] Derossi D, Calvet S, Trembleau A, et al. (1996). Cell internalization of the third helix of the antennapedia homeodomain is receptor-independent. J Biol Chem 271:18188–93. 8663410 10.1074/jbc.271.30.18188

[CIT0015] Derossi D, Chassaing G, Prochiantz A. (1998). Trojan peptides: the penetratin system for intracellular delivery. Trends Cell Biol 8:84–7. 9695814

[CIT0016] Derossi D, Joliot AH, Chassaing G, Prochiantz A. (1994). The third helix of the Antennapedia homeodomain translocates through biological membranes. J Biol Chem 269:10444–50. 8144628

[CIT0017] Deshayes S, Plenat T, Charner P, et al. (2006). Formation of transmembrane ionic channels of primary amphipathic cell-penetrating peptides. Consequences on the mechanism of cell penetration. Biochim Biophys Acta 1758:1846–51. 17011511 10.1016/j.bbamem.2006.08.010

[CIT0018] Ding Y, Wang YZ, Zhou JP, et al. (2014). Direct cytosolic siRNA delivery by reconstituted high density lipoprotein for target-specific therapy of tumor angiogenesis. Biomaterials 35:7214–27. 24875759 10.1016/j.biomaterials.2014.05.009

[CIT0019] Doherty GJ, Mcmahon HT. (2009). Mechanisms of endocytosis. Annu Rev Biochem 78:857–902. 19317650 10.1146/annurev.biochem.78.081307.110540

[CIT0020] Dreaden EC, Alkilany AM, Huang X, et al. (2012). The golden age: gold nanoparticles for biomedicine. Chem Soc Rev 41:2740–79. 22109657 10.1039/c1cs15237h PMC5876014

[CIT0021] El-Andaloussi S, Jarver P, Johansson HJ, Langel U. (2007). Cargo-dependent cytotoxicity and delivery efficacy of cell-penetrating peptides: a comparative study. Biochem J 407:285–92. 17627607 10.1042/BJ20070507 PMC2049024

[CIT0022] Ernsting MJ, Murakami M, Roy A, Li SD. (2013). Factors controlling the pharmacokinetics, biodistribution and intratumoral penetration of nanoparticles. J Control Release 172:782–94. 24075927 10.1016/j.jconrel.2013.09.013 PMC3891171

[CIT0023] Fretz MM, Penning NA, Al-Taei S, et al. (2007). Temperature-, concentration- and cholesterol-dependent translocation of L- and D-octa-arginine across the plasma and nuclear membrane of CD34+ leukaemia cells. Biochem J 403:335–42. 17217340 10.1042/BJ20061808 PMC1874247

[CIT0024] Gandhi NS, Tekade RK, Chougule MB. (2014). Nanocarrier mediated delivery of siRNA/miRNA in combination with chemotherapeutic agents for cancer therapy: current progress and advances. J Control Release 194:238–56. 25204288 10.1016/j.jconrel.2014.09.001 PMC4254052

[CIT0025] Giljohann DA, Mirkin CA. (2009). Drivers of biodiagnostic development. Nature 462:461–4. 19940916 10.1038/nature08605 PMC3936986

[CIT0026] Gratton SEA, Ropp PA, Pohlhaus PD, et al. (2008). The effect of particle design on cellular internalization pathways. Proc Natl Acad Sci U S A 105:11613–8. 18697944 10.1073/pnas.0801763105 PMC2575324

[CIT0027] Green M, Ishino M, Loewenstein PM. (1989). Mutational analysis of HIV-1 Tat minimal domain peptides-identification of trans-dominant mutants that suppress HIV-LTR-driven gene-expression. Cell 58:215–23. 2752420 10.1016/0092-8674(89)90417-0

[CIT0028] Grimmer S, Van Deurs B, Sandvig K. (2002). Membrane ruffling and macropinocytosis in A431 cells require cholesterol. J Cell Sci 115:2953–62. 12082155 10.1242/jcs.115.14.2953

[CIT0029] Gupta B, Levchenko TS, Torchilin VP. (2005). Intracellular delivery of large molecules and small particles by cell-penetrating proteins and peptides. Adv Drug Deliv Rev 57:637–51. 15722168 10.1016/j.addr.2004.10.007

[CIT0030] Harush-Frenkel O, Altschuler Y, Benita S. (2008). Nanoparticle-cell interactions: drug delivery implications. Crit Rev Ther Drug Carrier Syst 25:485–544. 19166392 10.1615/critrevtherdrugcarriersyst.v25.i6.10

[CIT0031] Hayer A, Stoeber M, Ritz D, et al. (2010). Caveolin-1 is ubiquitinated and targeted to intralumenal vesicles in endolysosomes for degradation. J Cell Biol 191:615–29. 21041450 10.1083/jcb.201003086 PMC3003328

[CIT0032] Herce HD, Garcia AE. (2007). Molecular dynamics simulations suggest a mechanism for translocation of the HIV-1 TAT peptide across lipid membranes. Proc Natl Acad Sci USA 104:20805–10. 18093956 10.1073/pnas.0706574105 PMC2409222

[CIT0033] Herce HD, Garcia AE, Litt J, et al. (2009). Arginine-rich peptides destabilize the plasma membrane, consistent with a pore formation translocation mechanism of cell-penetrating peptides. Biophysical J 97:1917–25. 10.1016/j.bpj.2009.05.066 PMC2756373 19804722

[CIT0034] Heuser JE, Anderson RG. (1989). Hypertonic media inhibit receptor-mediated endocytosis by blocking clathrin-coated pit formation. J Cell Biol 108:389–400. 2563728 10.1083/jcb.108.2.389 PMC2115439

[CIT0035] Hewlett LJ, Prescott AR, Watts C. (1994). The coated pit and macropinocytic pathways serve distinct endosome populations. J Cell Biol 124:689–703. 8120092 10.1083/jcb.124.5.689 PMC2119947

[CIT0036] Hillaireau H, Couvreur P. (2009). Nanocarriers’ entry into the cell: relevance to drug delivery. Cell Mol Life Sci 66:2873–96. 19499185 10.1007/s00018-009-0053-z PMC11115599

[CIT0037] Hunt JF, Rath P, Rothschild KJ, Engelman DM. (1997). Spontaneous, pH-dependent membrane insertion of a transbilayer alpha-helix. Biochem 36:15177–92. 9398245 10.1021/bi970147b

[CIT0038] Iversen T-G, Skotland T, Sandvig K. (2011). Endocytosis and intracellular transport of nanoparticles: present knowledge and need for future studies. Nano Today 6:176–85.

[CIT0039] Jahn R, Sudhof TC. (1999). Membrane fusion and exocytosis. Annu Rev Biochem 68:863–911. 10872468 10.1146/annurev.biochem.68.1.863

[CIT0040] Jain RK, Stylianopoulos T. (2010). Delivering nanomedicine to solid tumors. Nat Rev Clin Oncol 7:653–64. 20838415 10.1038/nrclinonc.2010.139 PMC3065247

[CIT0041] Jiang W, Kim BYS, Rutka JT, Chan WCW. (2008). Nanoparticle-mediated cellular response is size-dependent. Nature Nanotech 3:145–50. 10.1038/nnano.2008.30 18654486

[CIT0042] Jiao CY, Delaroche D, Burlina F, et al. (2009). Translocation and endocytosis for cell-penetrating peptide internalization. J Biol Chem 284:33957–65. 19833724 10.1074/jbc.M109.056309 PMC2797166

[CIT0043] Joanne P, Galanth C, Goasdoue N, et al. (2009). Lipid reorganization induced by membrane-active peptides probed using differential scanning calorimetry. Biochim Biophys Acta 1788:1772–81. 19427300 10.1016/j.bbamem.2009.05.001

[CIT0044] Kaihatsu K, Janowski BA, Corey DR. (2004). Recognition of chromosomal DNA by PNAs. Chem Biol 11:749–58. 15217608 10.1016/j.chembiol.2003.09.014

[CIT0045] Kaplan IM, Wadia JS, Dowdy SF. (2005). Cationic TAT peptide transduction domain enters cells by macropinocytosis. J Control Release 102:247–53. 15653149 10.1016/j.jconrel.2004.10.018

[CIT0046] Karabadzhak AG, Weerakkody D, Wijesinghe D, et al. (2012). Modulation of the pHLIP transmembrane helix insertion pathway. Biophys J 102:1846–55. 22768940 10.1016/j.bpj.2012.03.021 PMC3328699

[CIT0047] Kawamoto S, Takasu M, Miyakawa T, et al. (2011). Inverted micelle formation of cell-penetrating peptide studied by coarse-grained simulation: importance of attractive force between cell-penetrating peptides and lipid head group. J Chem Phys 134:095103. 21385001 10.1063/1.3555531

[CIT0048] Kerr MC, Teasdale RD. (2009). Defining macropinocytosis. Traffic 10:364–71. 19192253 10.1111/j.1600-0854.2009.00878.x

[CIT0049] Kiss AL, Botos E. (2009). Endocytosis via caveolae: alternative pathway with distinct cellular compartments to avoid lysosomal degradation? J Cell Mol Med 13:1228–37. 19382909 10.1111/j.1582-4934.2009.00754.x PMC4496137

[CIT0050] Kratz F, Warnecke A. (2012). Finding the optimal balance: challenges of improving conventional cancer chemotherapy using suitable combinations with nano-sized drug delivery systems. J Control Release 164:221–35. 22705248 10.1016/j.jconrel.2012.05.045

[CIT0051] Kumari S, Swetha MG, Mayor S. (2010). Endocytosis unplugged: multiple ways to enter the cell. Cell Res 20:256–75. 20125123 10.1038/cr.2010.19 PMC7091825

[CIT0052] Lee MT, Hung WC, Chen FY, Huang HW. (2005). Many-body effect of antimicrobial peptides: on the correlation between lipid's spontaneous curvature and pore formation. Biophys J 89:4006–16. 16150963 10.1529/biophysj.105.068080 PMC1366966

[CIT0053] Luzio JP, Parkinson MDJ, Gray SR, Bright NA. (2009). The delivery of endocytosed cargo to lysosomes. Biochm Soc Trans 37:1019–21. 10.1042/BST0371019 19754443

[CIT0054] Ma DX, Shi NQ, Qi XR. (2011). Distinct transduction modes of arginine-rich cell-penetrating peptides for cargo delivery into tumor cells. Int J Pharm 419:200–8. 21843610 10.1016/j.ijpharm.2011.08.001

[CIT0055] Mailander V, Landfester K. (2009). Interaction of nanoparticles with cells. Biomacromolecules 10:2379–400. 19637907 10.1021/bm900266r

[CIT0056] Matsuzaki K, Yoneyama S, Murase O, Miyajima K. (1996). Transbilayer transport of ions and lipids coupled with mastoparan X translocation. Biochem 35:8450–6. 8679603 10.1021/bi960342a

[CIT0057] Mellman I. (1996). Endocytosis and molecular sorting. Annu Rev Cell Dev Biol 12:575–625. 8970738 10.1146/annurev.cellbio.12.1.575

[CIT0058] Mellman I, Fuchs R, Helenius A. (1986). Acidification of the endocytic and exocytic pathways. Annu Rev Biochem 55:663–700. 2874766 10.1146/annurev.bi.55.070186.003311

[CIT0059] Mercer J, Helenius A. (2009). Virus entry by macropinocytosis. Nat Cell Biol 11:510–20. 19404330 10.1038/ncb0509-510

[CIT0060] Milletti F. (2012). Cell-penetrating peptides: classes, origin, and current landscape. Drug Discov Today 17:850–60. 22465171 10.1016/j.drudis.2012.03.002

[CIT0061] Mu QS, Hondow NS, Krzeminski L, et al. (2012). Mechanism of cellular uptake of genotoxic silica nanoparticles. Part Fibre Toxicol 9:29. 22823932 10.1186/1743-8977-9-29 PMC3479067

[CIT0062] Musial-Siwek M, Karabadzhak A, Andreev OA, et al. (2010). Tuning the insertion properties of pHLIP. Biochim Biophys Acta 1798:1041–6. 19766589 10.1016/j.bbamem.2009.08.023 PMC2862812

[CIT0063] Nichols B. (2003). Caveosomes and endocytosis of lipid rafts. J Cell Sci 116:4707–14. 14600257 10.1242/jcs.00840

[CIT0064] Nielsen PE, Egholm M, Berg RH, Buchardt O. (1991). Sequence-selective recognition of DNA by strand displacement with a thymine-substituted polyamide. Science 254:1497–500. 1962210 10.1126/science.1962210

[CIT0065] Pan R, Xu W, Ding Y, et al. (2016). Uptake mechanism and direct translocation of a new CPP for siRNA delivery. Mol Pharm 13:1366–74. 26937821 10.1021/acs.molpharmaceut.6b00030

[CIT0066] Parton RG, Del Pozo MA. (2013). Caveolae as plasma membrane sensors, protectors and organizers. Nat Rev Mol Cell Biol 14:98–112. 23340574 10.1038/nrm3512

[CIT0067] Parton RG, Howes MT. (2010). Revisiting caveolin trafficking: the end of the caveosome. J Cell Biol 191:439–41. 21041440 10.1083/jcb.201009093 PMC3003311

[CIT0068] Parton RG, Joggerst B, Simons K. (1994). Regulated internalization of caveolae. J Cell Biol 127:1199–215. 7962085 10.1083/jcb.127.5.1199 PMC2120257

[CIT0069] Pouny Y, Rapaport D, Mor A, et al. (1992). Interaction of antimicrobial dermaseptin and its fluorescently labeled analogs with phospholipid-membranes. Biochem 31:12416–23. 1463728 10.1021/bi00164a017

[CIT0070] Prestegard JH, Obrien MP. (1987). Membrane and vesicle fusion. Annu Rev Phys Chem 38:383–411.

[CIT0071] Ray A, Norden B. (2000). Peptide nucleic acid (PNA): its medical and biotechnical applications and promise for the future. FASEB J 14:1041–60. 10834926 10.1096/fasebj.14.9.1041

[CIT0072] Reshetnyak YK, Andreev OA, Lehnert U, Engelman DM. (2006). Translocation of molecules into cells by pH-dependent insertion of a transmembrane helix. Proc Natl Acad Sci USA 103:6460–5. 16608910 10.1073/pnas.0601463103 PMC1435408

[CIT0073] Reshetnyak YK, Segala M, Andreev OA, Engelman DM. (2007). A monomeric membrane peptide that lives in three worlds: in solution, attached to, and inserted across lipid bilayers. Biophys J 93:2363–72. 17557792 10.1529/biophysj.107.109967 PMC1965453

[CIT0074] Riehemann K, Schneider SW, Luger TA, et al. (2009). Nanomedicine-challenge and perspectives. Angew Chem Int Ed 48:872–97. 10.1002/anie.200802585 PMC4175737 19142939

[CIT0075] Rodrigues M, De La Torre BG, Andreu D, Santos NC. (2013). Kinetic uptake profiles of cell penetrating peptides in lymphocytes and monocytes. Biochim Biophys Acta 1830:4554–63. 23707662 10.1016/j.bbagen.2013.05.020

[CIT0076] Rothbard JB, Jessop TC, Lewis RS, et al. (2004). Role of membrane potential and hydrogen bonding in the mechanism of translocation of guanidinium-rich peptides into cells. J Am Chem Soc 126:9506–7. 15291531 10.1021/ja0482536

[CIT0077] Saalik P, Elmquist A, Hansen M, et al. (2004). Protein cargo delivery properties of cell-penetrating peptides. A comparative study. Bioconjugate Chem 15:1246–53. 10.1021/bc049938y 15546190

[CIT0078] Sarkar K, Kruhlak MJ, Erlandsen SL, Shaw S. (2005). Selective inhibition by rottlerin of macropinocytosis in monocyte-derived dendritic cells. Immunology 116:513–24. 16313365 10.1111/j.1365-2567.2005.02253.x PMC1802442

[CIT0079] Savic R, Eisenberg A, Maysinger D. (2006). Block copolymer micelles as delivery vehicles of hydrophobic drugs: micelle-cell interactions. J Drug Target 14:343–55. 17092835 10.1080/10611860600874538

[CIT0080] Shi NQ, Qi XR, Xiang B, Zhang Y. (2014). A survey on ‘Trojan Horse’ peptides: opportunities, issues and controlled entry to ‘Troy’. J Control Release 194:53–70. 25151981 10.1016/j.jconrel.2014.08.014

[CIT0081] Skotland T, Iversen T, Torgersen M, Sandvig K. (2015). Cell-penetrating peptides: possibilities and challenges for drug delivery *in vitro* and *in vivo*. Molecules 20:13313–23. 26205056 10.3390/molecules200713313 PMC6332435

[CIT0082] Swanson JA. (2008). Shaping cups into phagosomes and macropinosomes. Nat Rev Mol Cell Biol 9:639–49. 18612320 10.1038/nrm2447 PMC2851551

[CIT0083] Thevenin D, An M, Engelman DM. (2009). pHLIP-mediated translocation of membrane-impermeable molecules into cells. Chem Biol 16:754–62. 19635412 10.1016/j.chembiol.2009.06.006 PMC2741147

[CIT0084] Torchilin VP, Rammohan R, Weissig V, Levchenko TS. (2001). TAT peptide on the surface of liposomes affords their efficient intracellular delivery even at low temperature and in the presence of metabolic inhibitors. Proc Natl Acad Sci USA 98:8786–91. 11438707 10.1073/pnas.151247498 PMC37513

[CIT0085] Underhill DM, Goodridge HS. (2012). Information processing during phagocytosis. Nat Rev Immunol 12:492–502. 22699831 10.1038/nri3244 PMC5570470

[CIT0086] Van Kerkhof P, Sachse M, Klumperman J, Strous GJ. (2001). Growth hormone receptor ubiquitination coincides with recruitment to clathrin-coated membrane domains. J Biol Chem 276:3778–84. 11042179 10.1074/jbc.M007326200

[CIT0087] Verma A, Uzun O, Hu YH, et al. (2008). Surface-structure-regulated cell-membrane penetration by monolayer-protected nanoparticles. Nat Mater 7:588–95. 18500347 10.1038/nmat2202 PMC2684029

[CIT0088] Vives E, Brodin P, Lebleu B. (1997). A truncated HIV-1 Tat protein basic domain rapidly translocates through the plasma membrane and accumulates in the cell nucleus. J Biol Chem 272:16010–7. 9188504 10.1074/jbc.272.25.16010

[CIT0089] Wender PA, Galliher WC, Goun EA, et al. (2008). The design of guanidinium-rich transporters and their internalization mechanisms. Adv Drug Deliv Rev 60:452–72. 18164781 10.1016/j.addr.2007.10.016 PMC2533582

[CIT0090] Wijesinghe D, Engelman DM, Andreev OA, Reshetnyak YK. (2011). Tuning a polar molecule for selective cytoplasmic delivery by a pH (Low) insertion peptide. Biochemistry 50:10215–22. 22029270 10.1021/bi2009773 PMC3229090

[CIT0091] Wu CH, Chen YP, Wu SH, et al. (2013). Enhanced non-endocytotic uptake of mesoporous silica nanoparticles by shortening the peptide transporter arginine side chain. ACS Appl Mater Interfaces 5:12244–8. 24261815 10.1021/am4039882

[CIT0092] Xiang SN, Tong HJ, Shi Q, et al. (2012). Uptake mechanisms of non-viral gene delivery. J Control Release 158:371–8. 21982904 10.1016/j.jconrel.2011.09.093

[CIT0093] Yang J, Bahreman A, Daudey G, et al. (2016). Drug delivery via cell membrane fusion using lipopeptide modified liposomes. ACS Cent Sci 2:621–30. 27725960 10.1021/acscentsci.6b00172 PMC5043431

[CIT0094] Zaro JL, Shen WC. (2003). Quantitative comparison of membrane transduction and endocytosis of oligopeptides. Biochem Biophys Res Commun 307:241–7. 12859946 10.1016/s0006-291x(03)01167-7

[CIT0095] Zaro JL, Shen WC. (2005). Evidence that membrane transduction of oligoarginine does not require vesicle formation. Exp Cell Res 307:164–73. 15922736 10.1016/j.yexcr.2005.02.024

[CIT0096] Zhang Y, Li L, Han M, et al. (2015). Amphiphilic lipopeptide-mediated transport of insulin and cell membrane penetration mechanism. Molecules 20:21569–83. 26633348 10.3390/molecules201219771 PMC6332136

[CIT0097] Ziegler A. (2008). Thermodynamic studies and binding mechanisms of cell-penetrating peptides with lipids and glycosaminoglycans. Adv Drug Deliv Rev 60:580–97. 18045730 10.1016/j.addr.2007.10.005

